# Assessing the interplay between travel patterns and SARS-CoV-2 outbreak in realistic urban setting

**DOI:** 10.1007/s41109-020-00346-3

**Published:** 2021-01-13

**Authors:** Rohan Patil, Raviraj Dave, Harsh Patel, Viraj M. Shah, Deep Chakrabarti, Udit Bhatia

**Affiliations:** 1 Discipline of Computer Science and Engineering, Indian Institute of Technology, Gandhinagar, India; 2 Discipline of Civil Engineering, Indian Institute of Technology, Gandhinagar, India; 3 Discipline of Mechanical Engineering, Indian Institute of Technology, Gandhinagar, India; 4 King George Medical University, Lucknow, India

**Keywords:** Transportation network, SARS CoV2, Social networks, Transportation gravity models

## Abstract

**Background:**

The dense social contact networks and high mobility in congested urban areas facilitate the rapid transmission of infectious diseases. Typical mechanistic epidemiological models are either based on uniform mixing with ad-hoc contact processes or need real-time or archived population mobility data to simulate the social networks. However, the rapid and global transmission of the novel coronavirus (SARS-CoV-2) has led to unprecedented lockdowns at global and regional scales, leaving the archived datasets to limited use.

**Findings:**

While it is often hypothesized that population density is a significant driver in disease propagation, the disparate disease trajectories and infection rates exhibited by the different cities with comparable densities require a high-resolution description of the disease and its drivers. In this study, we explore the impact of creation of containment zones on travel patterns within the city. Further, we use a dynamical network-based infectious disease model to understand the key drivers of disease spread at sub-kilometer scales demonstrated in the city of Ahmedabad, India, which has been classified as a SARS-CoV-2 hotspot. We find that in addition to the contact network and population density, road connectivity patterns and ease of transit are strongly correlated with the rate of transmission of the disease. Given the limited access to real-time traffic data during lockdowns, we generate road connectivity networks using open-source imageries and travel patterns from open-source surveys and government reports. Within the proposed framework, we then analyze the relative merits of social distancing, enforced lockdowns, and enhanced testing and quarantining mitigating the disease spread.

**Scope:**

Our results suggest that the declaration of micro-containment zones within the city with high road network density combined with enhanced testing can help in containing the outbreaks until clinical interventions become available.

## Introduction

Modern history is witness to several infectious disease pandemics which have shaped our knowledge of their epidemiology, transmission, and management (Shearer et al. 2020). In the past 200 years, at least four strains of influenza, seven waves of cholera, tuberculosis, and the human immunodeficiency virus (HIV) have accounted for the deaths of nearly 100 million people (Cobey 2020; Egedesø et al. 2020). Mathematical modelling of infectious diseases allows us to predict an epidemic accurately, recognize uncertainties, and quantify situations to identify possible worst-case situations that can guide public health planning and decision making (Longini 1988; Grassly and Fraser 2008).

Classically, mathematical models of infectious diseases were dependent on the classification of individuals on their epidemiological status based on their potential ability to host and transmit a pathogen: Susceptible, Infectious, and Recovered [SIR] (Keeling et al. 2001; Keeling and Danon 2009; Anderson and May 1991). The SIR model is the most fundamental epidemiological model that relies on calculating the proportional burden of each of these three classes, and the transitions between them. In the context of an epidemic, assuming no births or deaths in a population, the only two possible transitions are infection (movement from susceptible to infected) and recovery (movement from infected to remission). For the sake of simplicity, it can be assumed that the susceptibility is proportional to the prevalence of infection or the disease burden in the community, and recoveries occur at a constant rate (Begon et al. 2002). Estimating transmission and recovery, the epidemic progresses exponentially until the growth rate slows and the epidemic curve plateaus; following which eventually over time the epidemic cannot be sustained and is eradicated (Keeling et al. 2001). A failing of the traditional SIR model is its inability to account for spatial aspects of disease spread. The 2001 dynamics of foot and mouth disease in the United Kingdom were demonstrated by explicit individualised modelling as transmission between farms that would not have been possible by traditional SIR models alone. Such models pointed at localised depletion of susceptible contacts as the mechanism for the slowing of the epidemic (Keeling et al. 2001; Ferguson et al. 2001). The HIV pandemic is characterised by a chronic infective state. The transmission of such a sexually transmitted infection is dependent on the host immune status, the infected individual’s viral load, and multiple social aspects like sexual practices of interactions between multiple structured risk groups within the population. In such a situation, the variability in infectious state predicts the progression of the epidemic and stochasticity merits more complex modelling (Wearing et al. 2005). Similarly, pandemics of influenza or flu-like illnesses can be accounted for by age and risk-structured models, which are explained by distinct risk groups for infection and fatality (age, health care workers, comorbid conditions), and the inherent nature of people to preferentially socialise with others of a similar age—a principle known as assortativity (Keeling and Danon 2009; Lloyd-Smith et al. 2003). More complex modelling focuses on the individual as a unit of the population describing individual interactions of each person in a population, in contrast to estimating the proportions of people with a certain disease status. The shift from a population-based model to an individual-based model is a powerful tool that helps to account for complex biologically and socially relevant interactions (Riley 2007; Ferguson et al. 2006).

Reliability of projections from individual based epidemiological model critically depends upon the realistic estimates of human mobility, which is often simulated using suite of agent-based simulations, network science approaches, and data science methods (Eubank et al. 2004; Salathé et al. 2010; Zhang et al. 2017). For example, Eubank et al. (2004) demonstrated the applicability of highly resolved agent based simulation tools that combine census and land use data with parameterized models to simulate the progression of infectious disease in realistic urban social networks (Eubank et al. 2004). Balcan et al. (2009) analyzed mobility data from various countries around the world and integrated in a worldwide structured meta-population mechanistic epidemic model to understand the role of infection due to multi-scale dynamic processes (Balcan et al. 2009). Ajelli et al. (2010) noted a good agreement between highly detailed agent-based modeling approaches and spatially structured mechanistic meta-population models. However, researchers note that while mobility networks used in meta-population models provide an accurate description of spreading phenomenon, detailed estimates of the impact at finer scales is hampered by the the low level of detail contained in such modeling schemes. On other hand, while agent-based modeling approaches are highly detailed, gathering high confidence detailed datasets, specifically in heterogeneous regions across the world, is a challenge (Ajelli et al. 2010). In the context of vector borne epidemics such as Zika virus (ZIKV) epidemic, researchers have integrated the high spatial and temporal resolution real-world demographic, mobility, socioeconomic and climatic condition datasets to estimate the profiles of ZIKV infections (Zhang et al. 2017). In other studies, researchers have combined the state-space models such as population based Suspected (S), Exposed (E), Infected (I), and Recovered/Removed (R) or SEIR models in combination with Google Trend datasets to understand the evolution of Flu trends in the United States (Dukic et al. 2012).When the SARS-CoV-2 epidemic spread across the Globe in early 2020, mathematical, statistical and machine learning based models gained prominence to find out how to slow and or stop the spread (Gilbert et al. 2020; Chinazzi et al. 2020; Kraemer et al. 2020; Vespignani 2020). Moreover, researchers referred to the ongoing efforts to model the spread of SARS-CoV2 as “war time”research where scientists have to deal with limited data, multiple assumptions and changing landscapes (Vespignani 2020). We note that despite the structural, mathematical and procedural differences which exist in the wide spectrum of epidemic models, realistic representation of human mobility patterns, and social interactions play a key role in governing model performance to understand the disease progression.

To model human mobility, travel demand models and activity based models are typically used. While travel demand models focus on estimating aggregate road usage in long run using aggregated zonal statistics often obtained from census and household surveys. More recent methods try to learn about human behavior in urban areas by using data collected from location-aware technologies including telecommunication activity datasets and Global Position System data archives (Jiang et al. 2016).We note that while massive and passive cellphone data can effectively generate complete urban mobility patterns (Hasan et al. 2013), these datasets are typically non-open source given privacy and security concerns. In absence of high resolution activity data, simplified models of human mobility such as gravity models are often used to understand the spreading of viruses and the evolution of epidemics (Ajelli et al. 2010; Mari et al. 2012; Li et al. 2011).

The novel Coronavirus (2019-nCoV) first identified in Wuhan, China has spread to 213 countries as of August 2020 with more than 24 Million confirmed cases reported worldwide (Dong et al. 2020). While much still needs to be learnt about the virus, its clinical characteristics, extent of inter-human transmission , and the spectrum of clinical disease, it is established that transmission of SARS-CoV-2 occurred to great extent through superspreading events (Perlman 2020). While accurate forecasting of spread as well as number of deaths and recoveries require ample historical data, contact networks, and identification of zeroth case in different regions in addition to the clinical parameters (Petropoulos and Makridakis 2020). While multiple modeling groups across the globes have proactively focused on modeling the spread at global and regional scales (Chinazzi et al. 2020; Vespignani 2020; Clark et al. 2020), studies at urban and city scales are limited (Minetto 2020; Tuite et al. 2020; Ribeiro 2020; Hâncean et al. 2020; Perc et al. 2020). Given the diversity in socioeconomic factors, spread characteristics, demographics, healthcare facilities, management of 2019-nCoV needs to involve local negotiations. Moreover, while stringent lockdowns and travel restrictions were imposed by many nations, compliance with such preventive measures varies considerably from one to another region (Wright 2020; Briscese 2020; Painter 2020). As a consequence, regions and cities with similar population densities and comparable socioeconomic indicators exhibited large disparities in the trajectory of the report SARS-CoV-2 cases.

In the present study, our objectives are two-folds. First, we attempt to understand the response of travel patterns in the city to partial lockdowns or creation of containment zones—zones which are declared as no travel zones due to widespread detected cases in a particular area. Using network science based node prioritization approaches adapted from (Bhatia et al. 2015)and travel demand models from (Ganin et al. 2017), we model the emergence of congestion zones within the city as a result of enforcement of containtment measures. We note that while creation of the containment zones are typically done to contain the spread of SARS-CoV-2, emergence of the congestion zones as a consequence of traffic rerouting could facilitate the disease spread.

Second, we attempt to understand the relationship between drivers of intra-city mobility and SARS-CoV-2 spread trajectory in different parts of the city. We use generalized stochastic SEIRS infectious disease dynamic model that allow us to model the effect of social contact network structures, heterogeneities, and interventions, such as social distancing, testing, contact tracing, and isolation on the spread trajectory of the disease. We hypothesize that in addition to the population density, ease of transit within smaller regions, which in turn is facilitated by the shorter transit/trip times, can result in accelerated spread of the SARS-CoV-2. We simulate the mobility patterns in different regions of the city of Ahmedabad which have disparate road network distribution and land use patterns but comparable population sizes. We select the period of nationwide lockdown as the temporal window for analysis. Since non-essential inter-district and interstate travel was prohibited during the period of lockdown, we simulate the travel patterns confined within the regions to mimic the population mobility for essential services.

**Fig. 1:**
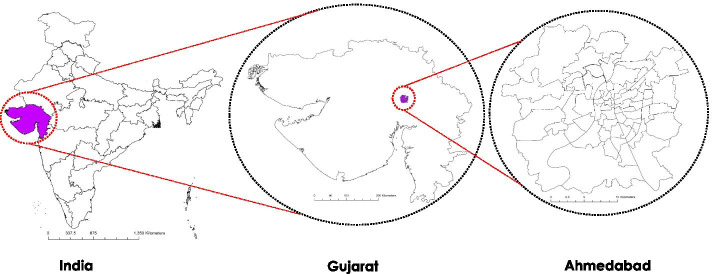
Study area of Ahmedabad Municipal Corporation (AMC), located in the central region of Gujarat in western India. Ahmedabad is selected as area of study given its distinction as financial hub of Gujarat, disparate socioeconomic distribution and high population density

**Fig. 2:**
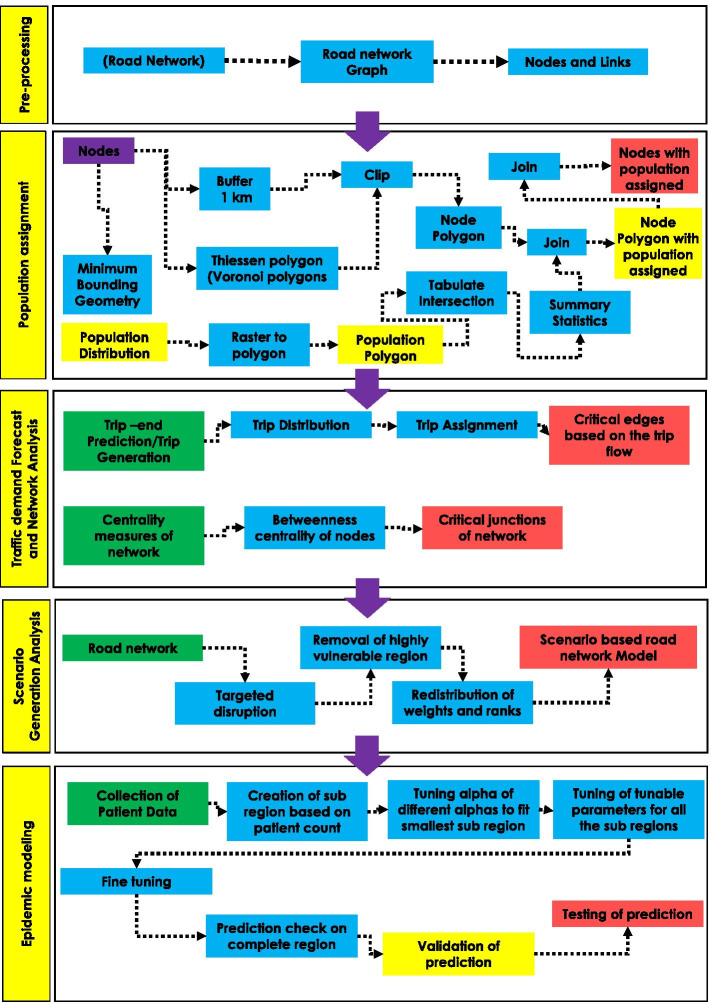
Overview of the modeling framework. The proposed methodology consists from top to bottom: Pre-processing incorporating the data collection and classification; Population assignment (Assignment of the population to each intersection); Traffic demand forecast and network analysis; Scenario generation analysis (Targeted disruption); and Epidemic spread modeling

## Region of study and data

We use Ahmedabad Municipal Corporation(AMC) as the study region (Fig. 1). Ahmedabad city has a population of 5.6 million (Census 2011), making it the fifth-highest populous city in India. Ahmedabad city witnessed a sudden increase in the patient count since the appearance of the first SARS-CoV2 case on March 19, 2020, and has been classified as one of the hotspot cities (Srivastava 2020). The flowchart of the methodology is shown in (Fig. 2). The Road data in the form of a shapefile is extracted from an open street map (OSM) data sets to generate a transportation network. India’s latest census data is from the year 2011, which may not provide an accurate result due to the duration gap. To address this, we considered the modeled population data of the year 2020 provided by the Worldpop with a spatial resolution of 30 arc-second (approximately 100m) (Tatem 2017). To understand the city’s traffic pattern, we use the Traffic Demand Analysis report, publicly available at (Corporation 2010). The SARS-CoV-2 patient time series data with location is collected from COVID India Tracker- an open-source repository that compiles the patient data from various government sources in near-real time (Group 2020).

## Methods and materials

### Geo-spatial boundary and population assignment

To assess commute patterns within the city, we rely on OpenstreetMap datasets and modeled population data obtained from Worldpop (Tatem 2017). We converted the road network into an undirected network graph, translating the road’s geometries into nodes and links. The attributes extracted from the data are the nodes’ location, length of links, categories of links, and the links’ end coordinates. The travel time of the road section is a crucial parameter to understand the behavior of traffic (Zheng et al. 2018). We calculate travel time considering the category (which guides speed limit) and length of a road section.

**Fig. 3:**
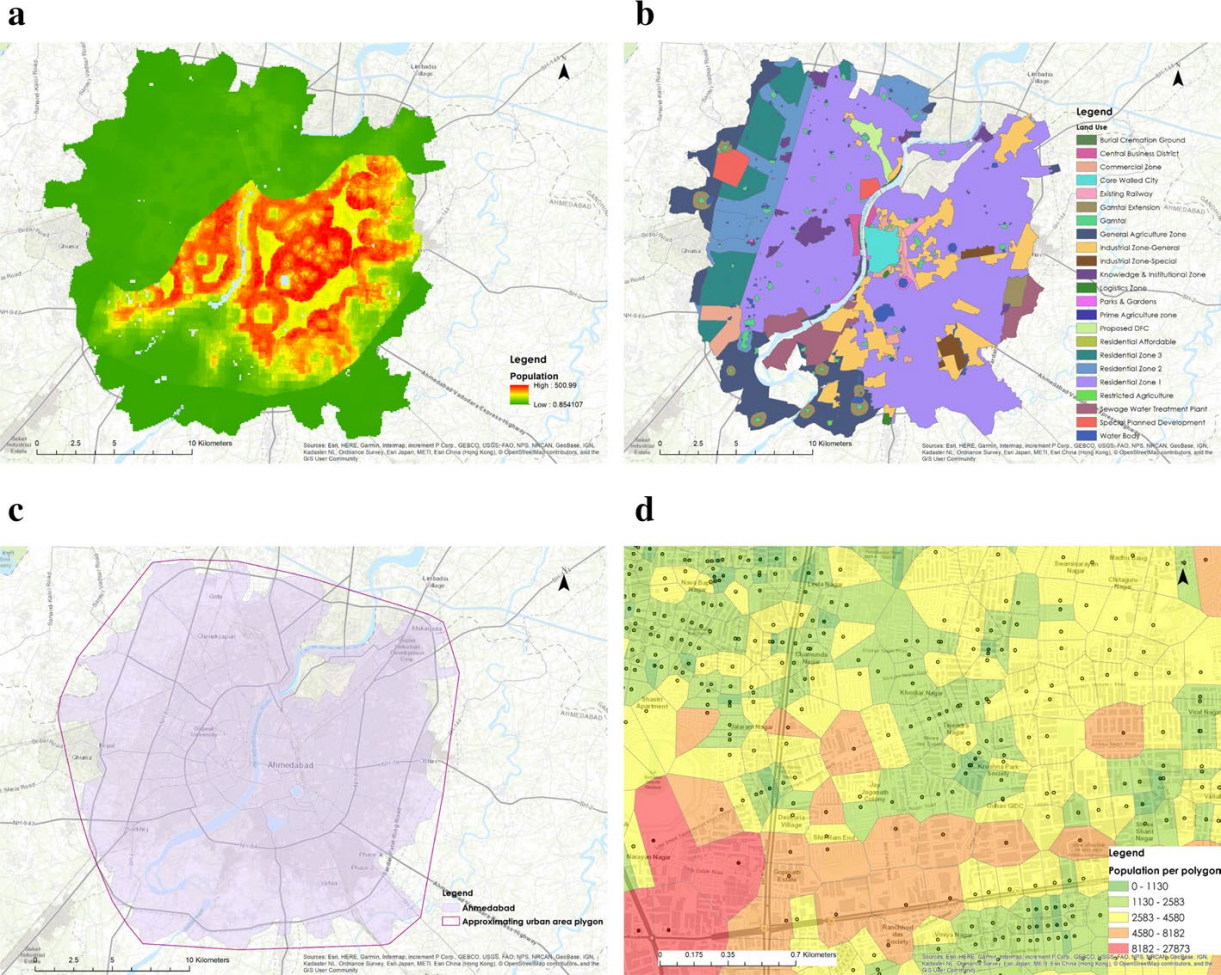
**a** Modeled population data extracted at 30 arc-second, **b** Land use distribution describing diverse arrangement of land use in the city, **c** Bound polygon to consider incoming and outgoing traffic and **d** Assignment of number of population served by intersection in a unique Voronoi polygons. Red colour is resemblance of high population, where as green colour is for low population

To define the transportation network boundary, we use the AMC boundary geospatial data and extracted the modeled population data for the same (Fig. 3a). To include the incoming and outgoing traffic from the city’s outskirts, we generated a buffer radius of 1 km. We considered the incoming traffic on the boundary intersections of the city network (Fig. 3c). Then the whole boundary of the city is split into a unique region of the Voronoi cell (Fig. 3d). we assigned the population to each voronoi polygon centered at intersections (Eq. 1). Each polygon population data is then transferred to their respective road intersections.

1 $$\begin{aligned} P_{i} = \Sigma \, P_{t} \times \frac{Area\,(C_{i}\cap C_{t})}{Area\,C_{t}} \end{aligned}$$

where:

- $P_{i}$ = Population in each Voronoi polygon
- $$P_{t}$$ = Modeled population
- $C_{i}$ = Cell of Voronoi polygon
- $C_{t}$ = Cell of modeled population

We also incorporate the land use (Fig. 3b) into the analysis to create real traffic congestion scenarios for post-lock down situations in the city. The assumption is made that around 1/4th of the population will commute from residential to commercial for daily work, making the industrial and commercial area more crowded than residential area. The re-allocation of population assignment help us identify the highly congested pockets in the city more realistically in absence of detailed data.

### Traffic demand forecasting model

We built a gravity-based transportation model to generate a commuter pattern that enables travel demand forecast while taking the population and household travel survey data into account. The gravity model is a classical trip distribution model widely used for trip distribution in an urban context (Casey 1955). The alternative approach to model the trip distribution are the queuing model (Shortle et al. 2018), growth factor model (Easa 1993), and activity-based model using human mobility patterns (Gonzalez et al. 2008). We discounted these alternative approaches as these models require extensive datasets, and it is difficult to get the data, especially in a lockdown scenario as the lockdown traffic patterns are completely different when compared to original traffic mobility.

We calculated the trip for road section based on the number of trips attracted to or leaving from specific zones. These trips are distributed by the linking of origin and destination, thus forming an Origin-Destination matrix. The allocation of these trips is done by considering the shortest possible route. We used the Djikstra algorithm (Dijkstra 1959) to find the shortest commuter distance (Eq.2)

2 $$\begin{aligned} T_{ij} = P_{i} \times \frac{\exp {(-aC_{ij})}P_j}{\sum _{(i,j) \in (V_S \times V_S)} P_j\exp {(-aC_{ij})}} \end{aligned}$$

where

- $T_{ij}$ = the number of trips produced in the zone i and attracted to zone j
- $V_S$ = the set of road intersections in the region *S*
- $P_i$ = the population of the $i^{th}$ road intersection
- $C_{ij}$ = the minimum time required to go from $i^{th}$ road intersection to $j^{th}$ road intersection.
- a = adjustable parameter to be fixed after model calibration

While deploying the gravity model, a fundamental assumption is “balancing” the trips generated and attracted. Here, balancing means that in a study area (an intersection), in our case, the total trips produced are equal to the total trips attracted to that intersection. The parameter used in the gravity model has been calibrated using the Household travel survey data in the study conducted by the Gujarat Industrial Development Board (GIDB) (Corporation 2010)

### Network analysis

To identify the high priority intersections and road segments within the transportation network of Ahmedabad, we use network centrality measures. These centrality provides a vital and widely used measure to analyze the network as it helps determine the most critical nodes in the network (Bhatia et al. 2015). In the context of road networks, betweenness centrality measures the extent to which an intersection lies on paths between other road segments. Intersections with the high betweenness may have considerable influence within a network by virtue of information/traffic flux that flows through to span across the network along the shortest path. Road networks include commuters’ flow along its edges and assuming that people tend to optimize their commute path by taking the shortest route. We calculated betweenness centrality for each intersection in the network and ranked according to this centrality value (Eq.3). The ranks demonstrate the intersection’s vulnerability in the network and demarcate the tendency for high connection with other intersections and can be considered as a measure for the potential for the intersection to develop into congestion points. We assigned the trip count calculated from the gravity model to each road section (links) and ranked them based on the possible transition. This provides the section with the highest vulnerability in terms of increased traffic flow.

3 $$\begin{aligned} Cb (v) =\sum _{u \in v} \sum _{w \in v/{u}}\frac{\sigma _{uw} (v)}{\sigma _{uw}} \end{aligned}$$

where

- $\sigma _{uw}$ = the number of shortest paths between u and w
- $\sigma _{uw} (v)$ = the number of shortest paths between u and w that passes v

### Scaling of disease spread

The primary factor influencing the disease spread is the population density (Tarwater and Martin 2001). The scaling of disease spread is computed with the population density and it helps to understand the nature of spread in an urban environment. This criterion holds when we do the extrapolation over the small region and there is uniformity in the community, which assumes that the social interaction is uniform (explained in supplementary for Interaction coefficient). However, this presumption falls apart on the city scale as the vast diversity in living conditions and connectivity contrast observed in different regions. To account for the disparate behavior, here we have considered the people’s interaction in a region is more if the population density is high and at the same time connectivity inside the region is competently developed. Based on the criteria mentioned above, we calculated the scaling factor to remove few limitations on the population density based extrapolation.The scaling factor for a region*S* with respect to another region $S_0$, $\kappa (S, S_0)$ is defined in (Eq. 4).

4 $$\begin{aligned} \kappa (S, S_0) = \frac{N_S \log {N_S}}{N_{S_0} \log {N_{S_0}}} \times \frac{A({S_0})}{A(S)} \times \frac{F({S_0})^2}{F(S)^2} \end{aligned}$$

- $N_S$ = Population of region *S*
- *A*(*S*) = Area of region *S*
- $$F(S)^2$$ = Interaction Coefficient

Here the F (S) is the independent interaction coefficient of the region and it is calculated using (Eq. 5).

5 $$\begin{aligned} F(S) = \sum _{(i,j) \in (V_S \times V_S)} \frac{\exp {(-C_{ij})}P_i P_j}{N_S^2} \end{aligned}$$

where

- $V_S$ = the set of road intersections in the region *S*
- $P_i$ = Working population at the $i^{th}$ road intersection
- $C_{ij}$ = the minimum time required to go from $i^{th}$ road intersection to $j^{th}$ road intersection in hours.

The route that gives the minimum time, $C_{ij}$ is calculated based on the assumed travel speed for disparate categories of roads. The complete calculation can be done using the transport network mentioned in the previous subsection. The value of *F*(*S*) is always less than 1 and the square of this value gives the interaction coefficient for the region *S*. The exact details for this construction are given in the supplementary section for interaction coefficient.

In the case of an isolated population, though population density in the region decreases at the same time, interaction time between the road intersection will increase, leading to an increase in the interaction coefficient (Eq. 5). This will produce the low scaling of disease spread as the isolated population area will be large and compare to the base region of the same number of population (Eq. 4).

The scaling factor we have defined in the Eq. 4 is a relative quantity between region *S* and $S_o$. To provide a single value that can be used to directly quantify multiple regions and to allow direct comparison between them, we use a base region for all calculations. The base region is the identity region *I*, which is not physically present but an imaginary construct such that $\frac{N_I log N_I}{A(I) F(I)^2} = 1$. Using this identity region as the base, for any two regions $S_1$ and $S_2$ (Equation 6)

6 $$\begin{aligned} \kappa (S_1, S_2) = \frac{\kappa (S_1, I)}{\kappa (S_2, I)} \end{aligned}$$

For notation purpose, we define $\kappa (S) = \kappa (S, I) = \frac{N_S log N_S}{A(S) F(S)^2}$

It is to be noted that the interaction factor is inversely proportional to the scaling factor. This is because the interaction factor does not consider the area of the region, which is separately considered in the calculation. The construction of the scaling factor is explained in a supplementary section for scaling factor.

**Fig. 4:**
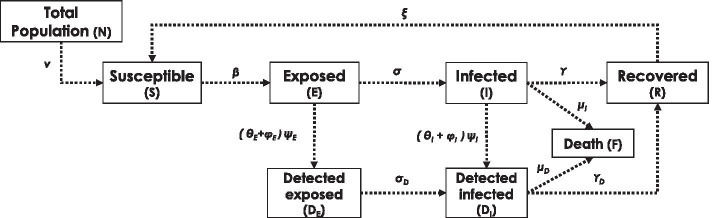
SEIRS plus compartment based model—the framework depicts the transition between disparate states

### SEIR PLUS model

SEIR PLUS epidemic spread model is applied to scaled-up data to predict the trajectory of spread. The SEIR model is a standard compartment based model. We used the dynamic form of the model in this study, which is used on stochastic dynamic networks (McGee 2017). The equations governing the state of the nodes is explained in (Eq.7). The conceptual framework of of compartment based SEIR PLUS model is shown in (Fig. 4).

7 $$\begin{aligned} Pr(X_i = S \rightarrow E)= & {} \left[ p\frac{\beta I}{N} + (1-p)\frac{\beta \sigma _{j\in C_G(i)} \delta _{X_j=I} }{|C_G(i)|} \right] \delta _{X_i=S}\nonumber \\ Pr(X_i = E \rightarrow I)= & {} \sigma \delta _{X_i=E}\nonumber \\ Pr(X_i = I \rightarrow R)= & {} \gamma \delta _{X_i=I} \nonumber \\ Pr(X_i = I \rightarrow F)= & {} \mu _I \delta _{X_i=I} \nonumber \\ Pr(X_i = R \rightarrow S)= & {} \eta \delta _{X_i=R} \end{aligned}$$

For testing purposes and quarantining, the above equations can be modified by adding some more compartments to account for quarantining and testing. The details are discussed in a supplementary section.

#### The clinical parameters

Parameters such as the rate of transmission, rate of progression, recovery rate, mortality rate depend on the cause of the epidemic. We obtained these parameters from the clinical data (Godio et al. 2020; Bagal et al. 2020). At the same time, there is also a dependency on the recovery rate when a positive person is detected early on or late.

We presumed that these parameters are independent of other factors related to social interaction and lockdown effects. A range was determined using available clinical data for such parameters, and fixed values from this range were used throughout. The values used for simulation are mentioned in Table 1.

**Table 1:** Values of parameters used for simulations

Clinical Parameter	$\beta$	$\sigma$	$\gamma$	$\beta _D$	$\sigma _D$	$\phi _I$	$\phi _E$	$\psi _E$	$\psi _I$
Values Used	0.187	5/26	1/15	0.155	5/26	0.4	0.8	0.04	1

#### Setting up the Scenarios

The interventions brought by the Government brings changes in the interaction of people compared to daily life. At the same time, a person detected positive will reduce the interaction with the rest of the population. To model the disparate scenarios, we use Erdős-Réyni graphs with different probabilities for edge creation. These probabilities are seen in the form $\alpha /N_S$ for a region *S*.

#### Tuning of parameters

The tunable parameters are the testing rates at different periods. Also, the SEIR model requires the number of initially infected people.

### Calibration of epidemic spread model

We now detail the steps followed during the calibration cycle:

- Pick a single sub-region, say $S^1_5$, and run the SEIR plus model with some arbitrary plausible values for the initial infected parameter, graph $\alpha$ values, and testing rates.
- Using the fitting between the observed data for infection and the resuts of the SEIR model to fine-tune the values to be used for testing rates and initial infected rates.
- At the same time, change the $\alpha$ parameters but keep them same across all regions.
- Check the output for all 10000 population subregions. Do further tuning of $\alpha$ values for the different garphs and also fine tune the testing parameters and initial infected for each region. It must be kept in mind that the initial infected are set only for 5000 population subregions and scaled values are used for 10,000 population subregions.
- Find a optimal way such that all plots fit with the observed values for all 5000 and 10,000 population subregions

**Fig. 5:**
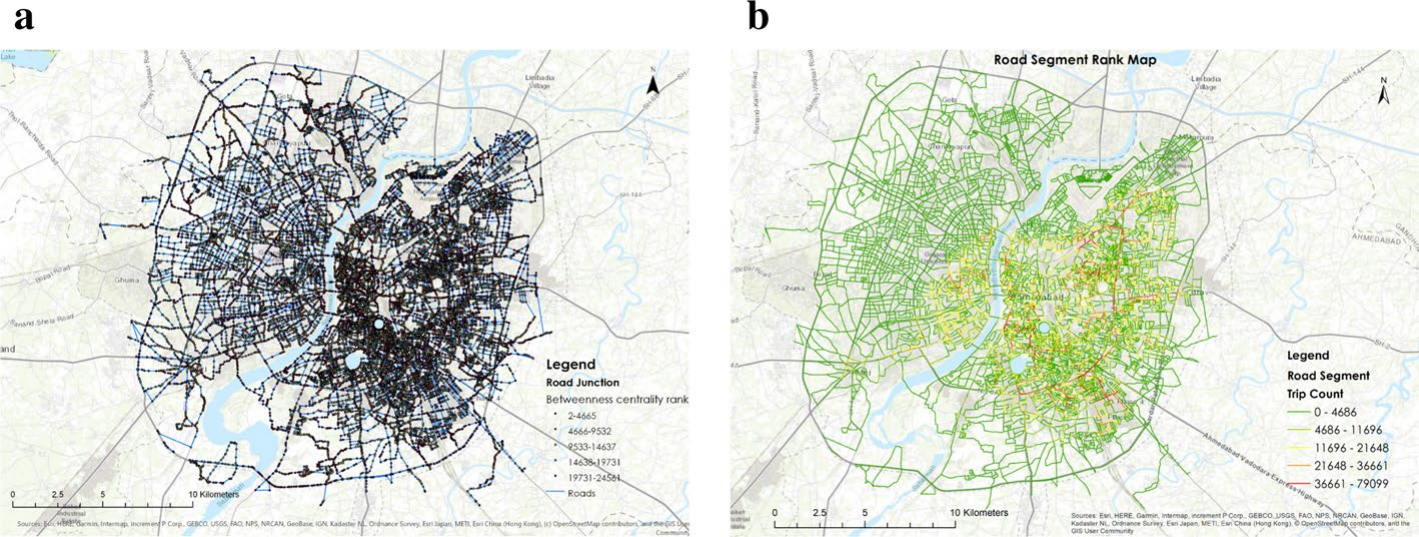
Maps of Ahmedabad city depicting the distribution of **a** Betweenness Centrality ranks across the road intersections. (red color nodes indicates high BC rank) **b** traffic congestion across the road links (red color road sections indicates higher congestion)

## Results

Generated gravity-based transportation model has two indicators: Betweenness Centrality rank (BC rank) of a road intersections (Fig. 5a) and probable trip count of each road section(Fig. 5b). These indicators help to identify densely connected pockets in the city. Through plotting these results, We have demarcated the possible traffic congestion pockets in the city. These results show the city pockets vulnerable to spread in post-lockdown scenarios. Our analysis shows that Ahmedabad’s central region has a high BC rank, demonstrating the priority intersection that can be vulnerable to disruption due to heavy traffic. The road segments with the high trip count are located in the city’s central and eastern parts, where population density is high. Also, it was seen that the first cases of COVID -19 were observed in this part of the city, making the transportation model useful while considering drive through testing.

**Fig. 6:**
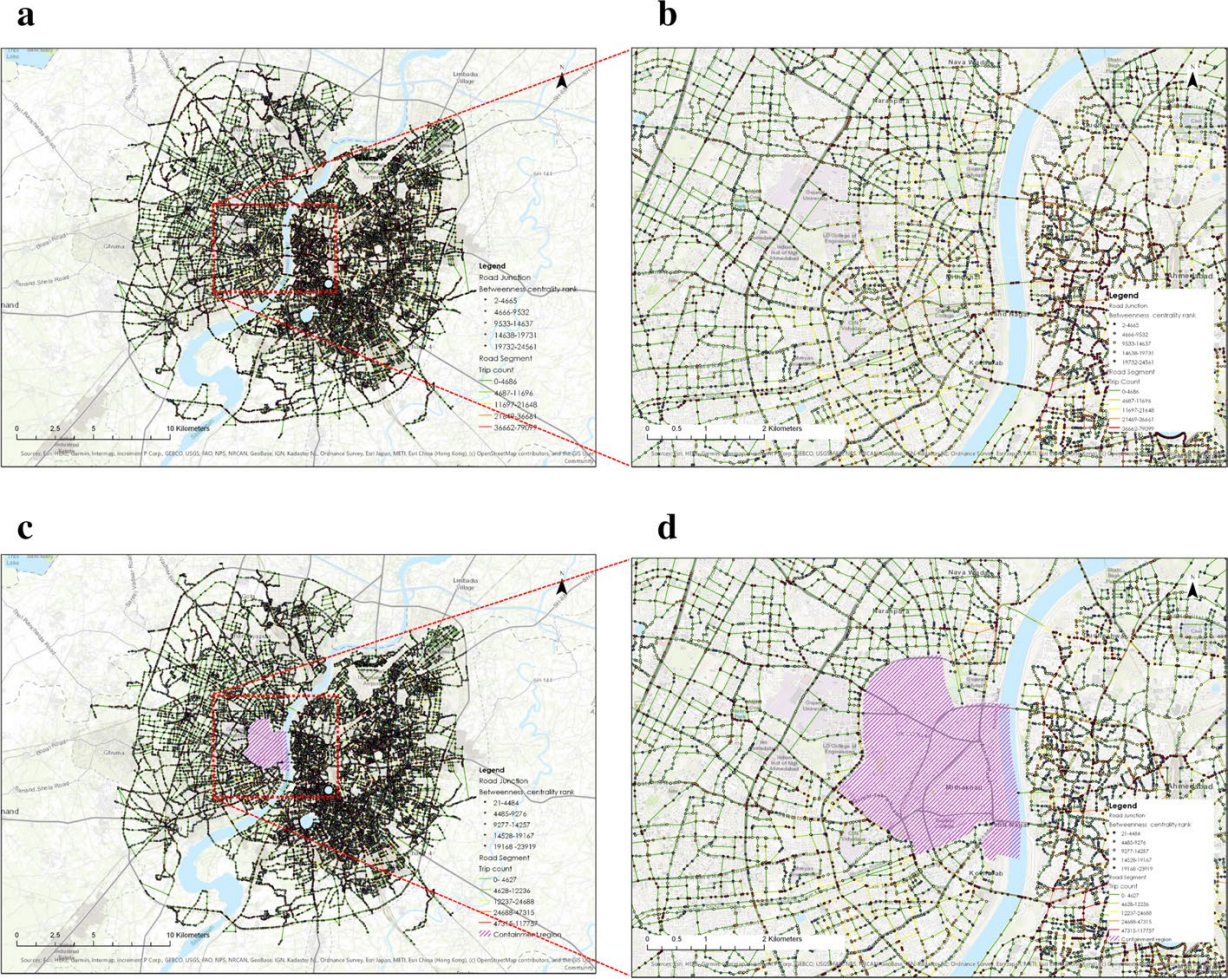
**a** The road network without disruption (normal condition), **b** Zoomed in map of road network without disruption, **c** Targeted disruption of road network to generate the lockdown scenario by removing containment zone and (SARS-CoV-2 spread situation) **d** Zoomed in map of targeted disruption

We simulated targeted disruption in the road network to analyze the system’s response, which resembles the often seen situations in the SARS-CoV-2 spread. Due to many cases in a particular region, the region might get quarantined or declared a containment zone. The containment zones make up the proper condition in network science that is termed as targeted disruption. The city network is witnessing the interruption in the traffic flow with the declaration of these containment zones as no travel zones. To analyze the disruption, we removed one of the city’s containment zones. The possible rerouting shows that this triggers the new potential vulnerable region to traffic congestion and spread through our re-calibrated indicators (Fig. 6).

**Fig. 7:**
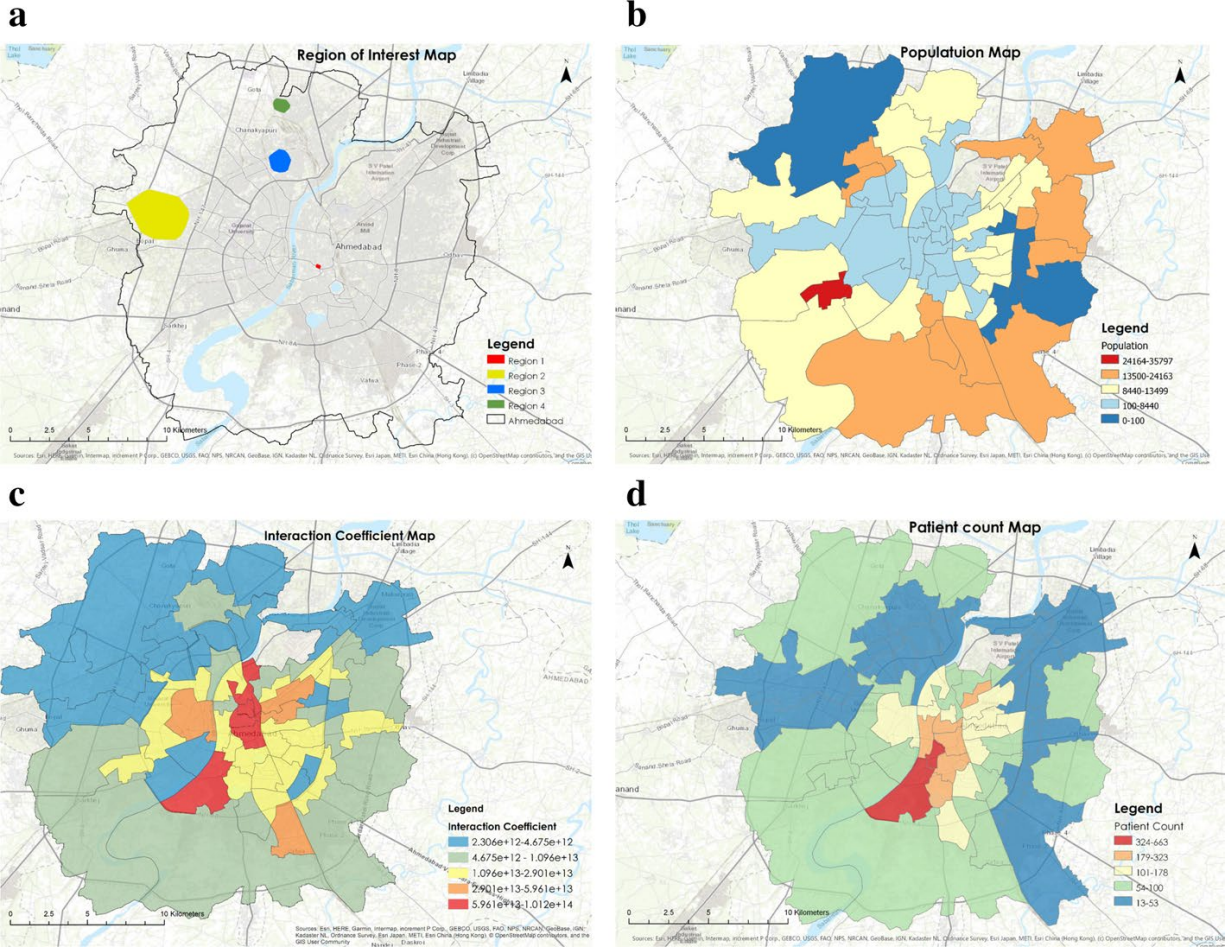
**a** Region of interest in the study area for epidemic spread model with same population, **b** population distribution in the city, **c** interaction coefficient distribution in the city depicting interaction ward wise and **d** SARS-CoV-2 Patient Distribution indicating the spread of disease in disparate regions

**Fig. 8:**
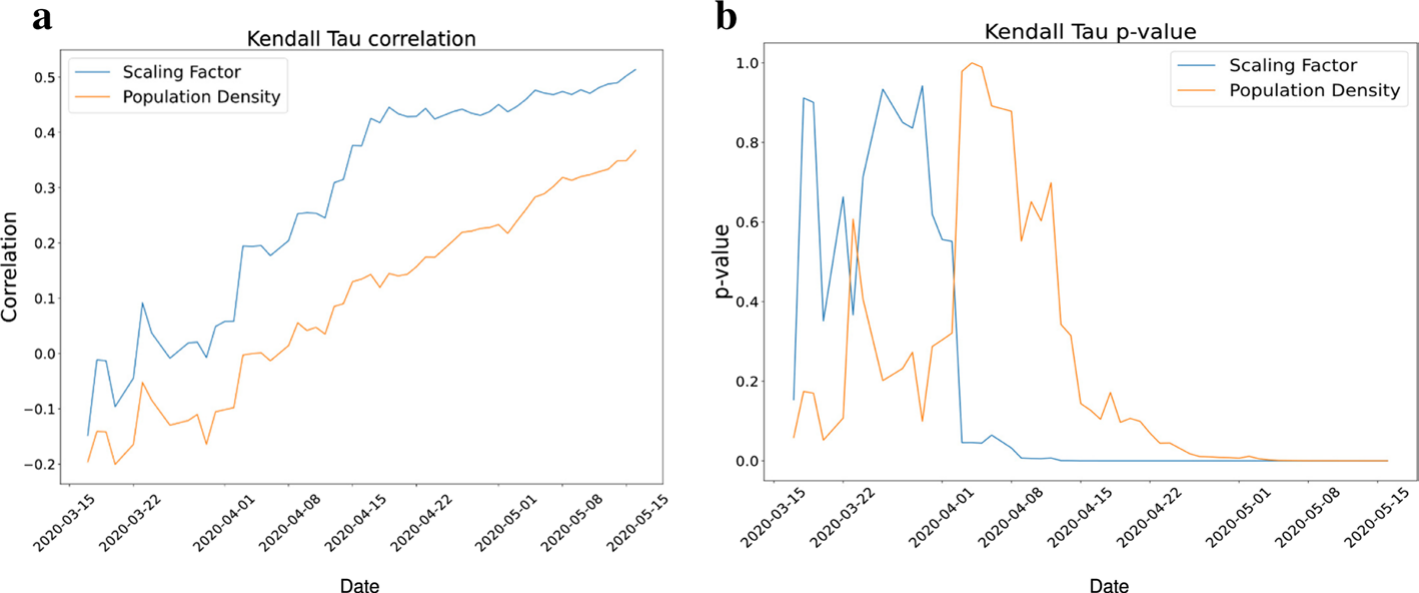
Ahmedabad city is divided into wards for administration purpose. **a** Shows the Kendall Tau Correlation of scaling factor and population density of wards with the number of cases **b** shows the p-value obtained for the correlation. From the plot, it is clear that scaling factor is highly correlated with the number of cases and that too with a very high confidence

The prediction of disease spread is difficult in the diverse living conditions and connectivity. In that case, we hypothesize that the scaling factor derived from the transportation network model provides a better alternative to population density as it considers the population density and the interaction between the community by road intersections. The travel time plays a crucial role in determining the scaling factor. To understand the importance of travel time, we have considered two regions with the same populations (Fig. 7a). The travel time changes with the number of intersections in the region. A lesser number of intersections in a region would translate to longer transit times, on an average, in an urban setting. This eventually leads to a decrease in the interaction coefficient and an increase in the scaling factor. Using this concept, the population density is calculated from population data (Fig. 7b) and the scaling factor through interaction coefficient (Fig. 7c) is calculated. Subsequently, we check the hypothesis by determining the Kendall–Tau Coefficient generated between the cumulative ward wise infected cases and the scaling factor and population density, respectively (Fig. 7d). This analysis validates our hypothesis and shows that the scaling factor provides a better correlation compared to the population density alone (Fig. 8a, b).

**Fig. 9:**
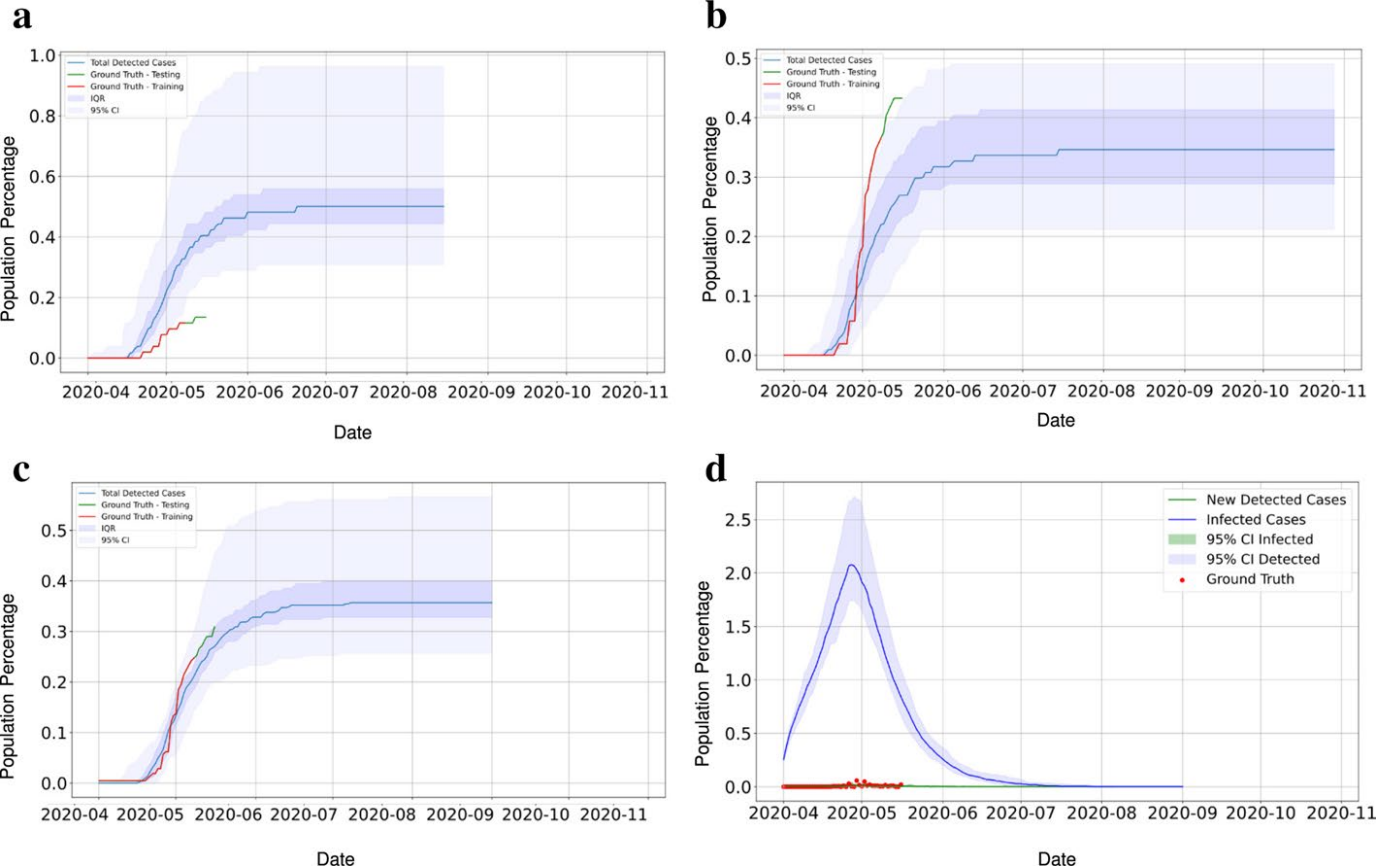
Region 1, shown in Fig. 7a has a population of 20000. Subregions of population 5000 and 10000 are selected from Region 1 where 5000 population subregion is inside the 10,000 population region: **a** Shows the total cases in 5000 population subregion. The training data of this subregion was used to tune the model parameters for Region 1, **b** shows the total cases in 10,000 population subregion. The training data of this subregion was used to do finer tuning of the model parameters for Region 1, **c** shows the total cases in Region 1. The training data was used to validate that the model is predicting properly. **d** Shows the prediction of infected cases and newly detected cases. The testing data was collected at a later date and used to check how well the model predictions match

**Fig. 10:**
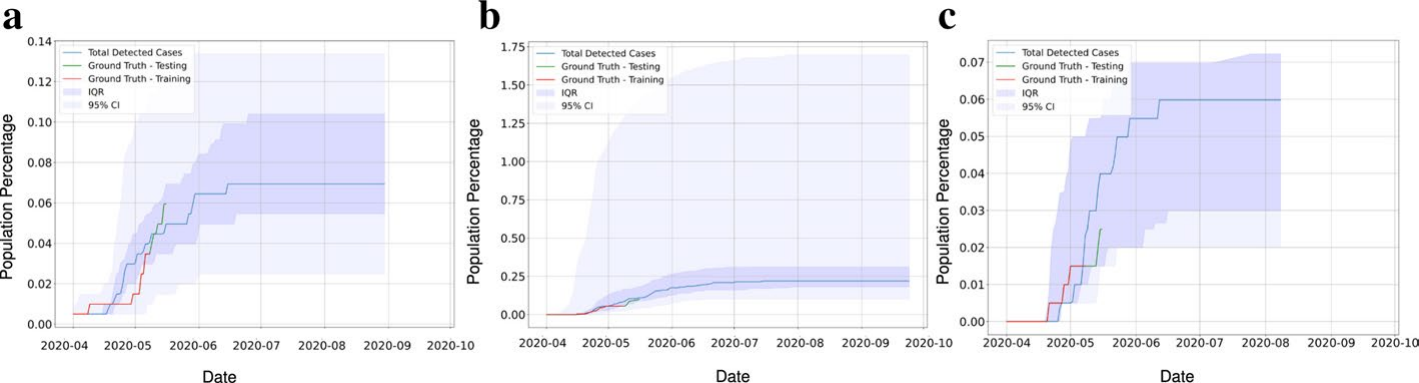
The three figures show the model prediction and the total cases in Region 2, 3 and 4 shown in Fig. 7a. All regions have approximately the same population of 20000. **a** Region 2 **b** Region 3 **c** Region 4. This figure shows that even though the population is the same, the spread trajectories and uncertainty bounds can be very different in each region

Once the hypothesis is tested, the calibrated clinical parameters are used to validate the prediction of the infected population for the regions. The epidemic SEIR plus model implemented on sub-regions with disparate ranges of population such as 5000, 10,000 and 20,000 is used to validate the model parameter values (Fig.  9a–c). This approach allows us to account for the variation that comes up with different issues of testing and variation in actual testings when only average testing rates are known. For region 1 with a 20,000 population, the SEIR plus model predicted the infected population shows a good agreement with the observed data. Region-1 shows the $\hbox {R}^{2}$, Relative Root Mean Square Error (RRMSE) and Relative Mean Absolute Error (RMAE) value as 0.9602, 0.688 and 0.558 respectively (Fig.  9d). The same approach we have implemented in region 2, region 3, and region 4, respectively (Fig.  10). We evaluate the prediction performance for the above-mentioned region and is shown in (Table 2). The value of $\hbox {R}^{2}$ is smaller in region 4 as the data for SARS-CoV-2 infected cases in the region was less than in other regions.

**Table 2:** Error matrix of prediction depiction the model performance on region of interests

Regions (20000 population)	$\hbox {R}^{2}$	RRMSE	RMAE
1	0.96	0.68	0.55
2	0.68	0.73	0.47
3	0.87	0.41	0.30
4	0.15	0.71	0.64

The SARS-CoV-2 spread has led governments to think of different interventions to reduce the spread by implementing various lockdown policies. These policies can change the trajectory of disease spread. To account this into action, we have also simulated the disparate policies in our model by selecting social distancing and relaxation combinations.

**Fig. 11:**
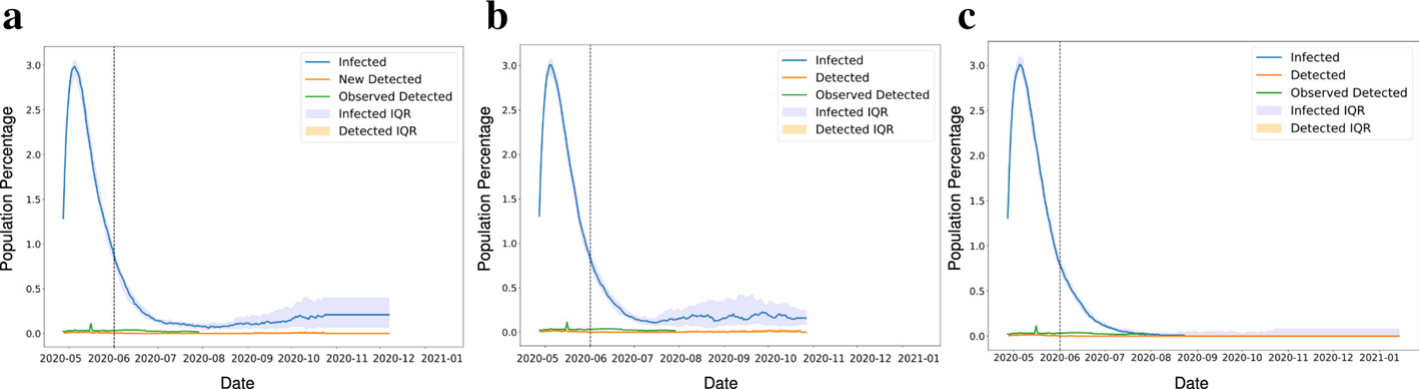
Time series data of patient count based on different lock down policies **a** Lock down relaxation by last week of May, unlock with the strict social distancing till mid June and then staged back to normal state in 50 days, **b** Lock down reduced by May end, unlock with the strict social distancing followed for 15 days and then staged back to normal state by mid July in staggered manner, **c** Lock down reduced by May end, unlock with the strict social distancing followed for 1 month and then staged back to normal state

The first policy considered is that if the government implements the lockdown relaxation by the last week of May 2020, unlock with the strict social distancing till mid-June 2020 and then staged back to a normal state in 50 days. The result shows that we can expect a sudden spike of cases in the initial days after lockdown relaxation. However, it gradually decreases in the upcoming months, but it shows the second wave of cases forming from September 2020 (Fig. 11a).

The second policy we consider is that the lockdown reduced by May 2020 end, unlock with the strict social distancing followed for 15 days and then staged back to normal state by mid-July 2020 in a staggered manner. The model result demonstrates an increased infected case count in the initial days after lockdown relaxation and shows the fluctuation in the infected cases in September and October 2020 (Fig. 11b).

Lastly, the third policy simulated is that the lockdown reduced by May end, unlock with the strict social distancing followed for one month, and then staged back to a normal state. The result demarcates that there is a rise in cases in the initial few days, but after that, from the month of July-2020, it can drop down very low (Fig. 11c).

## Discussion

The prediction of disease spread at the city scale is often complex due to diverse regional factors and data limitations. The road networks are the prime sources of intracity movements. This movement pattern can lead us towards the possible spreading of disease as most of the infectious diseases spread through social interactions. During these scenarios, prediction of disease spread through network-based epidemic spread models can be very helpful. This study has presented the unique approach to model the disease spread through transportation networks. The result of this analysis allows several meaningful inferences, which can make a high impact on the prediction of disease spread. First, this approach can be implemented in any congested city to determine the interaction between the population and lead to model disease spread. Most of the data considered during the modeling are open source or readily available. Second, framework also provides the flexibility of understanding disparate scenarios such as containment zone restriction. The third inference gained from the analysis is that the different lockdown policies are highly influenced social interaction and can be analyzed through the network-based epidemic spread modeling. Through this models policymakers can choose the best possible way to contain the spread. Although the interaction will vary from city to city based on local conditions, we anticipate that the overall patterns will be similar for comparable population densities and road networks. We further note that the quality, quantity and frequency of epidemiological datasets play an important role in establishing the correlations that we have observed in this study. While the proposed framework can be generalized to other cities, future efforts in this direction can greatly benefit from real-time mobility data obtained from cellphone activity or GPS data, and high-resolution clinical and epidemiological data with relatively longer duration of record.

## Supplementary information

**Additional file 1**. Supporting Information on Interaction Coefficient and Scaling Factor.

## Data Availability

Modeled Population data is collected from https://www.worldpop.org/. Road dataset is collected from https://www.geofabrik.de/data/shapefiles.html. Traffic demand Data is available through http://www.gidb.org/. Epidemiological datasets are available through https://api.covid19india.org/. Sourcecode of the epidemiological model used in this study is available at: https://github.com/ryansmcgee/seirsplus

## Abbreviations

2019-nCOV: 2019 novel coronavirus
AMC: Ahmedabad municipal corporation
BC rank: Betweenness centrality rank
HIV: Human immunodeficiency virus
OSM: Open street maps
RRMAE: Relative root mean absolute error
RRMSE: Relative root mean square error
SEIR PLUS: Susceptible, exposed, infectious, and recovered PLUS
SEIR: Susceptible, exposed, infectious, and recovered/removed
SIR: Susceptible, infectious, and recovered
ZIKV: Zika virus
