# Supplementary material for: Assessing the interplay between travel patterns and SARS-CoV-2 outbreak in realistic urban setting
Source: Appl Netw Sci. 2021 Jan 13;6(1):4. doi: 10.1007/s41109-020-00346-3 (PMC7803387; doi:10.1007/s41109-020-00346-3)
Supplement: Supplementary file 1 — Additional file 1. Supporting Information on Interaction Coefficient and Scaling Factor. [file 41109_2020_346_MOESM1_ESM.pdf]

**Supplementary Materials**

Interaction coefficient

We constructed the interaction coefficient based on the gravity model. The interaction between the two intersections is determined based on the two parameters: The first one is the fraction of the population in the individual intersections and the second is the time required to travel from one intersection to another. $C_{ij}$ gives the information of the time needed by a person while taking the best path possible for travel.

$C_{ij}$ is considered zero when $i$ and $j$ intersection are the same as the average time required for the interaction between two persons is assumed to be very small. This holds when $C_{ij}$ is measured in terms of hours or days. This assumption breaks when the measurement is in smaller units as the intra-intersection interaction cannot be approximated to $0$. Furthermore, if unit of $C_{ij}$ is large then a group of close-by intersections will behave as a single intersection as now even the interaction between them will start to get approximated to zero.

Considering the constraint mentioned above, the calculation of $C_{ij}$ is done in terms of hours. The selection of larger units such as day or month is not considered in the analysis because for the intersection in close proximity, the travel time will become negligible, leading to a single intersection. In the city environment, a person can reach any part of the city and nearby places in the time interval of hours. The unit of time for $C_{ij}$, in a sense, defines the resolution at which the system is being observed. If we consider that there is a single intersection in the region $S$, it is trivial to see that $F(S) = 1$. In the case of two intersections, the value of $F(S)$ will decrease as $C_{ij} \neq 0$ for all terms. The more skewed the distribution of population in the region, the smaller is the independent interaction coefficient.

$F(S)$ only considers the independent interaction between two intersections. But a commuting person may have to go from one intersection to another always through some other intersection. For example, consider that there are $3$ intersections in a region $S$.( Equation 8)

$$F(S) = \alpha_1^2 + \alpha_2^2 + \alpha_3^2 + 2(c_{12}\alpha_1\alpha_2 + c_{23}\alpha_2\alpha_3 + c_{13}\alpha_1\alpha_3) \tag{8}$$

where $\alpha_i$ is the fraction of population for $i^{th}$ intersection and $c_{ij} = \exp(-C_{ij})$.
considering (Equation 8) the interaction coefficient is

$$\begin{aligned}
F(S)^2 &= \alpha_1^4 + \alpha_2^4 + \alpha_3^4 + 4(c_{12}^2\alpha_1^2\alpha_2^2 + c_{23}^2\alpha_2^2\alpha_3^2 + c_{13}^2\alpha_1^2\alpha_3^2) \\
&\quad + 4(c_{12}\alpha_1^3\alpha_2 + c_{23}\alpha_1^2\alpha_2\alpha_3 + c_{13}\alpha_1^3\alpha_3) \\
&\quad + 4(c_{12}\alpha_1\alpha_2^3 + c_{23}\alpha_2^3\alpha_3 + c_{13}\alpha_1\alpha_2^2\alpha_3) \\
&\quad + 4(c_{12}\alpha_1\alpha_2\alpha_3^2 + c_{23}\alpha_2\alpha_3^3 + c_{13}\alpha_1\alpha_3^3) \\
&\quad + 8(c_{12}c_{23}\alpha_1\alpha_2^2\alpha_3 + c_{12}c_{13}\alpha_1^2\alpha_2\alpha_3 + c_{13}c_{23}\alpha_1\alpha_2\alpha_3^2) \\
&= \alpha_1^4 + \alpha_2^4 + \alpha_3^4 + 4(c_{12}^2\alpha_1^2\alpha_2^2 + c_{23}^2\alpha_2^2\alpha_3^2 + c_{13}^2\alpha_1^2\alpha_3^2) \\
&\quad + 4(\alpha_1^2 + \alpha_2^2 + \alpha_3^2)(c_{12}\alpha_1\alpha_2 + c_{23}\alpha_2\alpha_3 + c_{13}\alpha_1\alpha_3) \\
&\quad + 8c_{13}\alpha_1\alpha_2\alpha_3(\alpha_2 + c_{12}\alpha_1 + c_{23}\alpha_3)
\end{aligned} \tag{9}$$

The last term merges and accounts for the path that is passing through the 2$^{nd}$ intersection. If we extend it to $n$ intersections, there will be many cross-terms that will be extra. Also, there will be terms like $c_{12}c_{34}$, where the question comes that how did a person jump from 2$^{nd}$ intersection to 3$^{rd}$ intersection. A person may not always go by the shortest path but take small detours in the way. Suppose the shortest path from 1$^{st}$ intersection to 4$^{th}$ was direct, but the person went in 1-2-3-4 path. Then a single cross term cannot represent this information and the cross-terms with jumps account for this to some extent.

Calculating the population's interaction considering multiple paths that can be taken between two is an exponentially tricky question to solve. Taking the square of the independent interaction coefficient provides a simple approximation for the same.

Scaling Factor

The inverse proportionality of the scaling factor to the interaction coefficient comes because it captures the skew in the interaction in a fixed population of fixed area. Suppose we consider two regions with the same population and area; one with a single intersection and one with two intersections. For simplicity, in the second region, assume that the individual intersections are associated with half the area. In that case, if there is some travel time between them, then we cannot say that the population is using half of the area in the region. The population are using less area than that and this depends solely on the travel time given that both intersections have the same population. This indicates that more people are packed in the smaller region and the overall interaction will fall, while the the intra-intersection interaction will be more and the scaling of spread will increase.

Similarly, suppose only one intersection contains all the population and there is a significant travel time between the two intersections. In that case, this implies that most people are packed inside a small region, leading to a large decrease in interaction factor and a shoot in the scaling factor. This odd behavior can also be seen from the fact that the interaction factor normalizes on the basis that every node in a region has internal interaction proportional to the square of its population fraction. To correctly scale with the area and population of the region, $\kappa(S)$ is multiplied with $\frac{N_S}{A(S)}$ which is the population density of the region.

According to the Erdős-Réyni model for random graphs, the hard threshold for the connectedness of a graph $G(n, p)$ is $\frac{\log n}{n}$. The graphs mentioned in (Setting up the Scenarios section) will require the $\log N_S$ factor for scaling the probability correctly for the graphs as interaction coefficient and population density together quantify the net increase in connectedness of the random graph for that region.

SEIRS Plus with testing and quarantine

Two more compartments, $D_E$ and $D_I$, representing the detected exposed and detected infected, are added. To model the quarantine, a quarantine graph $Q$ is required, where the assumption is that detected people are in quarantine, and to model the testing, the parameters $\theta_E$, $\theta_I$, $\psi_E$ and $\psi_I$ are added, where $\theta_E$ and $\theta_I$ are the testing rates for exposed and infected people respectively and $\psi_E$ and $\psi_I$ are the rate of the positive test result for exposed and infected individuals respectively. The $\theta$ are tunable parameters, while $\psi$ are clinical parameters [54].

$$Pr(X_i = S \to E) = \left[ p\frac{\beta I + q\beta_D D_I}{N} + (1-p)\left( \frac{\beta \left[\sigma_{j\in C_G(i)}\delta_{X_j=I}\right] + \beta_D \left[\sigma_{k\in C_Q(i)}\delta_{X_k=D_I}\right]}{|C_G(i)|} \right) \right] \delta_{X_i=S} \tag{10}$$

$Pr(X_i = E \to I) = \sigma\delta_{X_i=E}$
$Pr(X_i = I \to R) = \gamma\delta_{X_i=I}$
$Pr(X_i = I \to F) = \mu_I\delta_{X_i=I}$
$Pr(X_i = R \to S) = \eta\delta_{X_i=R}$
$Pr(X_i = E \to D_E) = \left(\theta_E + \Phi_E \left[\sigma_{j\in C_G(i)}\delta_{X_j=D_E} + \delta_{X_j=D_I}\right]\right)\psi_E\delta_{X_i=E}$
$Pr(X_i = I \to D_I) = \left(\theta_I + \Phi_I \left[\sigma_{j\in C_G(i)}\delta_{X_j=D_E} + \delta_{X_j=D_I}\right]\right)\psi_I\delta_{X_i=I}$
$Pr(X_i = D_E \to D_I) = \sigma_D\delta_{X_i=E}$
$Pr(X_i = D_I \to R) = \gamma_D\delta_{X_i=I}$
$Pr(X_i = D_I \to F) = \mu_D\delta_{X_i=I}$
$Pr(X_i = any \to S) = \nu\delta_{X_i\neq F}$

Where:

- $\nu$: Rate of baseline birth
- $\mu_D$: Rate of infection-related mortality for detected cases
- $\sigma_D$: Rate of progression for detected cases
- $\gamma_D$: Rate of recovery for detected cases
- $\beta_D$: Rate of transmission for detected cases
- $\Phi_E$: Rate of contact tracing testing for exposed individuals
- $\Phi_I$: Rate of contact tracing testing for infected individuals
